# Nouveaux Elemens, &c. New Elements of Operative Medecine

**Published:** 1833-04-01

**Authors:** 


Nouveaux Elémens de Médecine Opératoire, accompagnés d'un Atlas de 20 Planches in 4° Gravées; représentant les principaux Procedés Opératoires et un Grand Nombre d'Instrumens de Chirurgie. Par *Alf. A. L. M. Velpeau.* Trois Tomes. Octavo, pp. 800—503—541. Paris et Londres, Baillière, 1832.

We take blame to ourselves for not having noticed this excellent work at an earlier period. It does credit not only to the author but the schools of France, and it places in a prominent point of view the advantages derived, and to be derived, from facilities in the acquisition of anatomical knowledge. This is really a treatise on operative surgery, such a treatise as should be in the hands of all surgeons, young and old. M. Velpeau regrets the taste for manuals and abridgements of all descriptions, short cuts to knowledge, or rather bye-ways to avoid it. We regret that taste, and we hope that the encouragement which M. Velpeau will receive, will prove that the reprehensible taste in question is not so universal as to preclude the success of excellent and comprehensive works.

We have taken occasion lately to point out the superiority of the French works upon anatomy, and on all more immediately connected with that science. Those who move in the scientific world acknowledge, we believe, that the French have carried the science of analysis to an extent that was not conceived attainable. Our scientific greatness is not of to-day. Reposing on our great men and the great things they have achieved, we have been in a complacent reverie for the last quarter of a century, and during that time the energy of France has enabled her to outstrip us. Wrapped up in our insular prejudices, and barricaded by our long war against all from without, we shut out the bird of science as well as the eagle of victory.

Another day has dawned upon us. Our mathematicians acknowledge the merits of the French, and are at this moment engaged in a generous rivalry that must benefit either, and the world. The demand for French and for German literature, the eagerness with which the works of imagination, of the belles-lettres, and of science, in those languages, are sought by intelligent and well-educated Englishmen, and the reciprocal desire so evident on the part of those nations to make themselves acquainted with our language and institutions, encourage the hope that a mental intercourse will take place between us, that will be equally serviceable, perhaps, to all.

Circumstances have made the French the European language; it is what Latin was. Our medical students should be acquainted with it, for, independently of the circumstance that what most gentlemen know should be known by our profession, they will find their means of obtaining information on points of medical and surgical science materially augmented. We fancy, however, that it is a work of supererogation to offer such advice.

We will not pretend to present a review of the work before us. It is in a great degree an elementary one, and although there is much that is justly and properly due to M. Velpeau, it is so interlaced with the more familiar matter, that it would be difficult and useless to attempt to separate it. Yet we think there are portions of the work to which we may usefully refer, and which may convey some information to such of our readers as have no opportunity of consulting the original.

In an introduction occupying 54 pages, M. Velpeau directs attention, amongst other things, to the preparations for an operation, and the circumstances to be considered during and after it. We need not say that such considerations, though much neglected, are of high importance, and we may possibly be excused for adverting to them a little more particularly.

M. Velpeau observes that the Spring and Autumn have been found upon the whole more favourable for operations, than the depth of Winter or height of Summer. Extremely hot weather is certainly unfavourable, for, independently of the greater tendency to suppuration, and of the inconveniences from the putrefaction of discharge, and so forth, there does appear to be a greater tendency in high states of temperature to severe forms of erysipelas or cellular inflammation. But, on the other hand, the variable weather of early Spring, and of the latter end of Autumn, would also seem to be favourable to the production of erysipelas, and it is usually at these seasons that erysipelas prevails in our hospitals. We have not the choice of season in the majority of cases. M. Velpeau observes that it is better, on the whole, to operate in the morning than in the evening, because we have the opportunity of paying attention to our patient when he chiefly wants it, at an early period after the operation; besides, he is less fatigued.

Moral precautions are of great consequence. All here depends on the tact of the individual. It is above all things necessary to inspire confidence, to make the patient go to the operating table with hope rather than distrust. M. Velpeau dwells upon the fact which has been often noticed, that those who screw their courage up to make no sound and betray no emotion whilst suffering under the knife, very frequently do badly afterwards. He observes that the surgeon should so reason with his patient before-hand, as to lead him neither to display unnatural and forced apathy, nor give way to excess of timidity. We believe that much may be done by consummate address on the part of the surgeon, but much also depends on the temperament of the patient, which is neither under his nor our control. We remember seeing an instance of this sort at the Military Hospital of Antwerp. A Dutch colonel, Koopman, was received there from the citadel. He was wounded in the right forearm, and in the lower extremities, by the explosion of a shell. The wounds were severe, but not excessively so. He was an immense, fat, leuco-phlegmatic man, and during the dressings he betrayed great irritability, impatience, and a sensitiveness to pain, which he endeavoured to smother. He appeared to feel his situation as a prisoner. Soon after the dressing there was hæmorrhage from the wound in the forearm. It was necessary to remove the dressings and to search for the vessel. He was now still more irritable and impatient. He died that evening of tetanus that supervened four hours after the dressing.

It would be highly important to ascertain some certain general rule, respecting the diet and medical treatment of patients, previous to their undergoing operations. This is not so simple as some might imagine. One person depletes his patient, starves, purges, bleeds him; another gives him merely a little less to eat; a third feeds him, (for we have witnessed this); a fourth does nothing at all. Who is right? We fancy that there can be no rule applicable to all or even to many cases. The surgeon must be guided by his judgment and experience. We are confident we have seen operations proceed unfavourably in consequence of diminishing the diet previous to and after the operation; and we are equally confident we have seen cases where more attention to the use of aperients, and of regulated diet would have been advisable. But we repeat that each case must be looked at per se—we must be guided by the peculiar nature of the operation, and more especially by the habits of the patient. If a patient has had a chronic disease for which he has had a generous diet, and if immediately before or immediately after the operation he be deprived of that diet, it seems to us, from what we have observed, that a greater tendency to suppuration is occasioned. On the whole, we suspect that it is better to sin on the side of non-interference than on the reverse.

M. Velpeau condemns the practice of crowding the operating part of the amphitheatre of Hospitals. There can be no doubt of its impropriety in every point of view. The choice of assistants is always one of importance. They ought not to be too numerous, but at all events the duties of each should be clearly and appropriately pointed out, and satisfactorily understood.

Nothing is more common than to witness, in a private operation, a great degree of confusion from inattention to this apparently trifling circumstance. All seem operators or spectators and none assistants. M. Velpeau also insists very earnestly on the full preparation and convenient disposition of all things necessary for the operation. The instruments, the bandages, &c. should be placed on a side table in the order in which they will be wanted, and if anything be taken up it should always be replaced in its proper place. Those accustomed to hospital operations know the absolute necessity of these precautions. M. Velpeau reprehends surgery by the stop-watch. The barbarity of *merely* attending to time, is, we hope, no longer dreamt of by even the youngest pupil. At the same time there is nothing more dreadful to bear or more painful to see borne, than a protracted clumsy operation.

Amongst the consequences of operations M. Velpeau notices at some length, and he could not avoid it, the secondary inflammations. Many, even now, consider these secondary inflammations as rather matters of curiosity than of much practical importance. There cannot be a more gross delusion. These secondary inflammations are of extreme frequency, and were we to generalise on what we have personally witnessed, we should say that they are the most frequent cause of death after operations, if not after accidents. For one patient who dies in a large London hospital of erysipelas, two or three would seem to die of the secondary inflammations. It is true that we have hitherto failed to discover any means of controlling these inflammations when established, and so far the investigation may appear to be unattended with practical results. But though we cannot cure, we may perchance prevent, and an accurate acquaintance with the phenomena exhibited by these inflammations, and a careful observation of the circumstances under which they do or do not occur, may possibly lead to measures of prevention of the utmost benefit.

We shall notice M. Velpeau's observations, and that on two accounts; first, that the subject, as we have said, is one of extreme importance, and cannot be too frequently brought before the profession; and, secondly, that having been led by the evidence of facts to modify opinions that we formerly entertained and expressed, we are not averse to take advantage of the present opportunity of correcting ourselves. It may signify nothing to many what such humble individuals as ourselves may think; but as we have reason to believe that this Journal exercises some little influence on the minds of the profession, more, perhaps, than we might have a right to expect, it becomes our duty as it is our inclination to endeavour to render the information it conveys as exact, and the opinions it presents as sound, as circumstances and our poor abilities will permit.

In the number of this Journal for October, 1830, we reviewed at some length Mr. Arnott's paper in the Medico-Chirurgical Transactions on the Secondary Effects of Inflammation of the Veins. At the close of that review will be found the following passage. "We cannot say that we think much of the translation of pus, the metempsychosis, as it were, of our modern pathologists. The deposites, whether visceral, articular, or seated in the cellular substance, are neither in quality nor quantity related to the secretion of the parent wound or injured part, and it appears to us extremely ridiculous to imagine that suppurations beat up their quarters and take to another more favoured spot in the body, like a tribe of Bedouins in search of a spring in the desert, or an Arab palace under the magic influence of an Aladdin's lamp! To attribute these curious affections 'to the very difficult subject of the pathology of the blood,' is a supposition that leaves us and them where we were, and we believe after all that the theoretical notions respecting the visceral deposites are not one whit the clearer for the present investigation." Bad as the wit of the foregoing passage may be, we believe that on the whole it is better than the reasoning. Since that time we have seen more facts and we hope we are something the wiser for them. As our only object is truth it gives us not the slightest mortification to acknowledge an error. Before we consider this question more fully we must pay our respects to M. Velpeau, and we are disposed to lay before our readers his summary of the phenomena of these affections, as being at once concise and complete.

M. Velpeau ranks together phlebitis and the secondary deposites, and we think justly. He does not, however, consider the former as the essential cause of the latter, and here again we agree with him. But of this bye and bye. Let us look at his description of the symptoms and march of these affections.

Sometimes the secondary inflammation begins by a violent fit of shivering which may last some hours—sometimes by mere horripilation—in some cases by merely a slight coldness of the extremities. We would add, that in many cases it commences with vomiting, unaccompanied by any perceptible cold stage, and we think this is more common when the cellular tissue is the seat of the deposite. The skin becomes pallid, assumes a yellowish tint, somewhat livid or blueish, and soon afterwards the aspect is more or less cadaverous. This sort of attack differs from that of intermittent fever, in the circumstance that the cold stage is rarely followed by decided reaction. If sweating succeeds it is unequal, often thick and clammy. We should say that, for the most part, the fever attending the formation of a deposite has a distinct cold, and a profuse sweating stage, whilst the intermediate hot stage is extremely imperfect. After one or more paroxysms, returning after irregular intervals, a typhoid condition commonly succeeds. The eyes sink in the orbits, the conjunctiva becomes yellow, and the lips pallid, and the whole face dirty-looking and sallow. The tongue is usually moist, and does not become encrusted till a late period of the disease. The pulse is frequent and hard, and becomes more and more weak. The belly becomes distended—sometimes there is diarrhœa—rarely delirium, but always a degree of oppression of the mental faculties. In some subjects vague symptoms of visceral inflammations become associated with the foregoing. A slight flush appears upon the cheeks, and may be accompanied with a little cough, or pain in the chest, or dyspnœa. Sometimes there is jaundice more or less decided, with pain or uneasiness in the region of the liver or in the right shoulder; or, which is unfrequent, some vomiting, redness and prominence of the extremity and borders of the tongue, which grows dry; or, finally, pains in some parts of the limbs, for instance, in a large joint. The thirst is not commonly excessive. The breath, always fetid, exhales sometimes a true purulent odour. We have remarked, as all must have done, a very peculiar and nauseous odour about the persons of these unfortunate patients; that odour has likewise been always present in the pus which their wound or wounds contained. During this time cicatrization is suspended in the wound, the edges of which are partially absorbed, and pale like the rest of the surface. Whatever was the character of the suppuration previously, it becomes of an unhealthy serous character. We would particularly direct attention to the state of the wound in these cases. It is highly characteristic. If there were granulations they become absorbed, so that there is a pale glistening surface on a level with, or below the surrounding parts; new skin formed at the edges of the sore is partially removed by ulceration, so that the border has a remarkably abrupt and irregular appearance. The discharge consists of flakes of pus, floating in serum, and having a peculiarly sickly odour. Such has been the state of the sore in very many of the cases we have witnessed, but it is right to state that this state of sore is chiefly seen in cases which are not rapidly fatal. Emaciation, to revert to M. Velpeau's description, makes progress, the muscles and bones are detached from each other, a bloody discharge escapes, and becoming more and more fluid, resembles at last the washings of flesh, and at length the patient dies exhausted, from the twelfth to the thirtieth or fortieth day.

There are two symptoms which have been but little adverted to in the foregoing description. The first is the remarkable frequency of the pulse which is almost invariably observed, at least has been so by us, in these cases. See the patient when we will, his pulse is seldom, if ever, below 100, often considerably above it. This is a circumstance of some importance, and we shall advert to it again hereafter. The next symptom is, that peculiar state of mind, which patients affected with the deposites usually exhibit. Whilst the organic system is evidently much disturbed, the circulation hurried, the functions deranged, nutrition annihilated, whilst death is in his face, the patient says that he is better, that he is well. We pass to the consideration of the cadaveric lesions.

These are found to be of different kinds, though referrible, says M. V. to the same cause. The most frequent are numerous purulent deposites in the proper cellular tissue of the viscera, or collections, more or less abundant, of greyish, creamy, or purulent serum, in the serous cavities. In other instances the large articulations are filled with pus, which is also found either in the state of depôt, or of infiltration, wherever there is a certain quantity of loose cellular membrane.

The arteries are almost empty, and the blood that they contain remains, in general, extremely fluid. The veins contain more, and the blood is more evidently altered. The coagula are many-coloured, black, yellow, white, greenish, and have a granular texture most distinct on cutting, or crushing them between the fingers. They sometimes contain globules of pus distinguishable by the naked eye, and it is not even uncommon to find collections of some little volume in the interior of the clots. M. Velpeau has seen these in the iliac, uterine, caval veins, and in the cavities of the heart, &c. Many of these concretions are still soft and recent, some firm, and apparently of a certain date, none, in most cases, connected with any particular morbid state of the vessel in which they are contained. In the neighbourhood of the wound nothing is more common than to see the veins inflamed, with suppuration within and without to a variable extent, though seldom to such as to involve the venæ cavæ.

To revert to the depôts, or insulated depositions of purulent matter. These have been found in all organs. A subject examined by M. Velpeau at Tours, in 1818, presented some dozens of them in the brain and in the tissue of the heart. A young man who died at the Clinique of the Faculty, in 1825, had them in the spleen and in the kidney. The liver and the lungs are the viscera most frequently affected. When once fairly recognized in these viscera, it seems hardly possible for the observer to confound them with other lesions, yet, strange to say, they have been so confounded, and there are many who so confound them still. Most frequently there are many in the same organ—they are mostly seen near the surface—they rarely acquire a large size, varying from that of a pin's head to that of a large nut or small egg. On pressure they are felt like tubercles in the viscus, and its surface is nodulated by those that are most superficial. In the liver they are more central, ordinarily larger than in other parenchymata, and more unequal in consistence: for they are often fluid in the centre and consistent at the circumference. The different phases of the deposite can be followed out more satisfactorily in the lung. In parts we distinguish only small spots resembling ecchymosis—when we advance, these ecchymoses contain each in the centre a little drop of pus—in other places, only purulent collections are observed. These deposites may be either concrete, or of different degrees of fluidity. The substance of some appears to be confounded with the surrounding tissues, to have penetrated, or been diffused through them; others again have cysts, the parietes of which are of a villous consistence, and lilac colour. Beyond these alterations the organ is natural, and the depôts are almost always separated by intervals of healthy tissue. In many instances, indeed, the organ appears to have been merely excavated to afford room for the deposites. We have frequently witnessed more inflammation of the tissue of the organ, more particularly of the lung, than M. Velpeau describes. It is not uncommon to observe considerable interlobular peripneumony, or even extensive inflammatory *engouement* of the lung. We have seen these deposites produce sloughing of the lung in their vicinity.

The effusions into the serous cavities are equally remarkable. The pleura is most commonly affected, though the peritoneum, pericardium, arachnoid may be so. The quantity of the effusion becomes in a few days very considerable. The membrane, scarcely altered in other respects, is covered with a layer of more or less consistence of true pus, whilst the liquid has an ashy or earthy tint. In the joints the tissues remain surprisingly sound, although they are filled with pus. The cartilages, however, may be partially destroyed, the synovial membrane and the ligaments ulcerated, without alteration of the contiguous parts. So it is with the subcutaneous depôts, or those in the cellular texture of the limbs. Yet occasionally there are traces of inflammation around these depositions.

Some patients present at once collections of matter in different parts and various organs. The majority have them but in one organ. Sometimes they exist in the lungs or liver, without effusion into the serous cavities—sometimes there is merely effusion into these cavities—sometimes they are only in the limbs, and they may be within or without the articulations—and, finally, patients die, if we understand M. Velpeau aright, with symptoms of this affection, but no cadaveric proof of it. He places the mischief in these cases in the blood. We may mention a curious fact on this head. In the old St. George's Hospital depôts were very frequently found in the liver; perhaps it was the most common seat of them. Since the erection of the new building the liver has been only affected in two or three instances, but the great majority of patients have had the depôts in the lungs.

We now pass to the causes of these affections, and we instantly feel that we are treading on treacherous and slippery ground. All hitherto has been the mere expression of facts; etiology is their analysis and value.

We come to the etiology of these affections. It is an error to suppose that they were unknown to our predecessors. They were aware of the fact that deposites of pus occurred in distant parts after injuries and operations, but the fact itself was ill observed and worse explained. We believe that the merit of directing the particular attention of modern surgeons to the subject, must chiefly be attributed to M. Velpeau, though the late Mr. Rose, Mr. Guthrie, Mr. Arnott, and others in this country, may fairly lay claim to a considerable share.

There are several opinions as to the causes of these depôts. One party supposes that they depend on the resorption of pus, with or without phlebitis—a second, that the resorption of pus is always connected with phlebitis—a third, that they are the result of simple idiopathic inflammations—a fourth, that they depend on some peculiar state of the nervous system. There may be other sects, but the four we have mentioned are the chief.

We need not argue the question of simple idiopathic inflammations. There are few who now entertain this opinion, and, consequently, it would be fighting with a shadow to combat it.

Is the state of the nervous system to be looked on as the cause? We were at first inclined to attribute great importance to the agency of the nervous system; we are now rather shaken in our belief. It must be confessed that it is difficult to reason satisfactorily on the agency of a system so complicated and so powerful, and it does appear to us extremely unphilosophical to get rid of that difficulty, by denying the influence of the nervous system altogether. It is not improbable that it does exercise an influence, a mysterious one indeed, in the production of the deposites. Thus we find that the patients most commonly affected with them, are persons who have lived and drunk freely, debauchees in whom the nervous system is extremely excitable, and who are subject to delirium tremens. When we think upon this fact, we cannot resist the suspicion that certain states of the nervous system are more favourable to the development of secondary inflammations than others. Beyond this, we know of no certain data for satisfactory conclusions.

If we are not content with the theory of nervous agency, we are necessarily driven to the humoral pathology. Is the blood then contaminated in these cases, and is pus absorbed or in any way carried into the current of the circulation? Let us look at facts.

Purulent depôts seldom if ever occur, unless there be a suppurating wound, or pus be locked up in some part of the body. The cases in which the secondary inflammations are most common are undoubtedly those of compound fractures, amputations, operations, in which the knife passes deeply among parts, injuries of the head, and inflammation of the veins. Wherever, after an injury or an operation, inflammation and formation of lymph or pus occurs in the intermuscular cellular tissue, a part in which it must *necessarily* be confined, we may count pretty generally on secondary inflammation occurring somewhere. This fact has struck us most forcibly, and so far as our own observation extends, we can guarantee its accuracy. If, on the other hand, suppuration occur where it is not of necessity locked up, for instance, in the subcutaneous cellular tissue from which incisions discharge it, the secondary inflammations are beyond comparison less common. This is a broad fact that carries with it extreme weight.

It may be said that in chronic abscesses, in what is termed the psoas abscess for example, pus is locked up for a length of time, without much tendency to secondary inflammations existing. The cases do not appear to us to be parallel, nor the analogy sufficiently exact to diminish the force of the previous fact. A psoas abscess is connected with a peculiar state of constitution, has altogether a different march, occasions very different symptoms, from those remarked in cases of deep cellular inflammation after a recent injury or operation. No two things can be more unlike in appearance, and in all essential circumstances, than a patient during the first week or two after the reception of a compound fracture, and one with chronic abscess. In the former the inflammation is a spreading one—in the latter the purulent collection is more or less bounded by a wall of lymph.

We repeat then that the cases in which we must peculiarly dread the supervention of secondary inflammations, are those in which the inflammation has occurred in parts, where pus, if secreted, must be necessarily confined. We need hardly say that such cases must be most adapted for the resorption of pus. But there is another circumstance that cannot fail to have weight. The cases in which we might imagine, à priori, that pus might pass with much facility into the circulation, are those of phlebitis. Now two facts present themselves to our notice:—first, that phlebitis is very commonly attended with the purulent depôts, and secondly, that the symptoms of phlebitis are so similar to those of the depôts, that, accidental circumstances of distinction not being present, we defy any surgeon to point out the difference. It will be observed that this does not involve the question of the existence of phlebitis in all cases of depôts. That is a totally separate consideration.

Facts, then, have carried us thus far; first, that depôts are most common where, after injuries or operations, matter is confined, and particularly where it is not lodged in any one cavity, but disseminated through the intermuscular cellular membrane; secondly, that phlebitis is very frequently accompanied or followed by the depôts; and, thirdly, that the leading symptoms occasioned by phlebitis and by the depôts are almost exactly similar.

We know not whether we have put the argument in a clear manner, or whether our readers see its force as we have seen the force of the facts it represents. Those facts have made us swerve from an inclination to believe in the efficient operation of the nervous system, to a more than suspicion that the true cause exists in the passage of pus, in one way or other, into the circulating system. The evidence is not perfect we allow, but it is of a cumulative character, and more satisfactory than that on the side of nervous agency.

We have said that the constant existence of phlebitis in cases of depôts requires other evidence for its support. The facts to which we have alluded do not prove it. We have seen many cases of secondary inflammation, in which a careful inspection detected no venous inflammation. This has been the case with other observers. M. Velpeau has examined, since this theory was broached, thirteen patients in whom he could discover no phlebitis whatever. Some of these dissections were made in the presence of those who adhered to the theory in question. It has been said that the smaller veins are affected, and that it is difficult to trace their implication. But this is obviously assumption, and where facts fail we must stop. When the evidence of sense no longer serves us, one man has, cæteris paribus, as good a right to deny as another has to affirm.

With reference, therefore, to the co-existence of phlebitis and the purulent deposites, it seems to us that we may venture on the following conclusions, but that facts do not yet support us farther. First, that the symptoms of phlebitis and the purulent deposites are very similar, if not identical in kind. Secondly, that in a considerable number of cases, phlebitis and the purulent deposites are found, by examination after death, to co-exist. Thirdly, that in many cases a very careful inspection can detect no traces of phlebitis, while the purulent deposites are present. Fourthly, that in many cases, phlebitis exists without the purulent deposites.

There is one circumstance that has always made a strong impression on our minds, and to which we cannot forbear adverting. It is this. If after an injury or an operation a patient has continued to have a very frequent pulse, that patient has almost always done ill, and has in many instances died of some secondary inflammation. A frequent pulse is, of course, merely an evidence of fever, and fever itself is usually a symptom, an assemblage of deranged functions from some local lesion or irritation. We do not mean to say that fever is never idiopathic, but we may safely say that in the cases we are treating of it seldom is so.

It appears then that if, after an injury or an operation, that description of fever which is characterized by a pulse always frequent, but more so at night, and a temperature of the surface always above par, be continued for any length of time, the patient is very liable to be attacked by some secondary inflammation. We are not solicitous about the explanation of facts, provided the facts themselves be true. Our own impression is, that if, after any local injury, fever from any cause be maintained, that very fever becomes itself the source of other affections. The blood is circulating and continues to circulate in all the organs with unusual rapidity, and it is not difficult to conceive that under such circumstances those organs should be more prone to inflammation. There are some analogies that bear upon this point. It is well known, for instance, that during the re-actions that follow any kind of collapse, visceral inflammations are not uncommon. Thus a patient receives a severe burn, there is much collapse, stimulants are given to a great extent, considerable re-action succeeds, and fatal inflammation of the pleura or peritoneum supervenes. Mr. Earle mentions that many of the firemen burnt during the conflagration of Covent Garden Theatre, died from visceral inflammations. They were much stimulated. Now in these cases the secondary inflammations occur in some instances before suppuration has commenced in the injured parts, and consequently before absorption of pus can occur. But persons labouring under intermittent fevers are liable to serous or pneumonic inflammations. In intermittents there is no cognizable source of pus to be absorbed. A very common cause of death in patients who are said to have typhus, is inflammation of the lung. We have seen a patient die of secondary pleuritis, who had simply cutaneous erysipelas, and in whom there was nowhere suppuration. These facts, and we might mention many more, facts too of such a nature that any man of experience or of observation must recognize them as not uncommon, are calculated, we think, to prove that consecutive inflammations occur under conditions, and in diseases agreeing only in this, that there is fever.

We repeat that we are not solicitous about an explanation, nor do we feel anxious respecting the fate of our *opinions.* All that we covet is the power of observing facts correctly, and drawing from them the fair inductions they allow. Were we inclined to press our theory, or rather generalization, for it scarcely deserves to be termed a theory, we might instance the ulcerations of the bowels, the chronic pleurisies, the tubercles even occurring in protracted febrile states of system, whether the pyrexia be occasioned by a suppurating burn, diseased bone, diseased joint, or in many other manners. We will, however, desist, contented with hinting our belief, that consecutive diseases, whether they be visceral inflammations rapidly fatal, or merely chronic, are apt to occur whenever pyrexia is present for any length of time, whatever the cause of that pyrexia may have been.

Our readers perhaps may not share our interest in this discussion. We pray them to pardon us if we have dwelt on it too long. We must say, however, that to us it seems extremely important, and we think it is our duty to lay before our readers as many authentic facts as possible. We pass to the treatment, and here, unfortunately, we have little that is satisfactory to dwell upon.

When public attention was first attracted to the consecutive inflammations, it was supposed that could depletion be resorted to very early in consequence of an improved diagnosis, it might prove beneficial. This reasonable expectation has not been fulfilled. In some cases there is more decided pain, more inflammatory action, than in others. In these, small general bleedings or local depletion are beneficial to a slight extent, that is, they relieve the more urgent symptoms. But they do no more, and in some instances they appear to precipitate the patient's fate. M. Velpeau seems to have arrived at the same conclusion. He speaks more favourably of purgatives and blisters, indeed we have witnessed more benefit from the latter than from any other means. M. Velpeau recommends the quina when there are intermissions. Tartar emetic has failed him.

We would observe that there are two kinds of secondary affections—one, in which there is much serous or visceral inflammation; another, in which there are depôts comparatively unattended with inflammation. It seems reasonable to suppose on general principles that these may admit of variations of treatment. In the former we should adopt local depletion, and especially blisters; in the latter it is that quina and the mineral acids protract the fatal termination. We saw the oxymuriate of mercury tried in one case; it did no good. We saw the nitric acid tried in another, with the same result. Mercury so pushed as to put the patient rapidly under its influence we have not seen employed.

The means of cure are confessedly deficient and inefficacious. A few patients would certainly seem to have survived, but it is doubtful what was the extent of mischief, and what the consequence of treatment. Our great aim should be directed to prevention.

If we are correct in inferring that the two great causes of secondary inflammations, are matter that does not get a free escape, and fever maintained in any manner, it follows of course that we should endeavour to avoid, or to remove them. With respect to the lodgment of matter we need say no more than to direct attention to the well-known principles and modes of practice in such circumstances. Matter, however, is frequently collected in the intermuscular cellular membrane when its existence is unsuspected. To enter on this subject would not suit us at present, but we hope to be able to offer our remarks on this affection at an early period. With regard to the second cause, fever, we can only recommend the surgeon to look to what produces it, and if possible to remove the agent. It is usually local mischief of some description. Occasionally the cause is of a more general nature. Thus, a patient accustomed to spirituous liquors, if suddenly deprived of them after an injury or an operation, is not unfrequently attacked with low fever, which passes away when his accustomed stimulant is given to him. On the other hand, we feel confident that we have seen surgeons sin on the other side, and, dreading this danger, run into the opposite of over-feeding and over-stimulating. Were we to trust our own suspicions and observations we should say, that over-feeding, and over-treating with bark and stimulants or tonics, is too frequently a cause of the depôts. The truth is, that all depends on the judgment of the surgeon in the management of the individual case. Of two, equally well-informed on the general principles of treatment, one shall be able to seize the indications of the case before him, and the other shall not; one, in fact, shall be a good surgeon and the other a bad one.

With respect to the local treatment, that which is unirritating, the absence of pressure, light dressings, with occasional fomentations, and the use of all means for the evacuation of matter, whether position, the laying open of sinuses, or making of dependent openings, or poultices, are the best and safest.

There are parts of M. Velpeau's work which we shall notice on fitting occasions hereafter.

